# Numerical correlation between non-visual metrics and brightness metrics—implications for the evaluation of indoor white lighting systems in the photopic range

**DOI:** 10.1038/s41598-023-41371-3

**Published:** 2023-09-08

**Authors:** Tran Quoc Khanh, Trinh Quang Vinh, Peter Bodrogi

**Affiliations:** 1 Department of Electrical Engineering and Information Technology, Laboratory of Adaptive Lighting Systems and Visual Processing, Technical University of Darmstadt, 64289 Darmstadt, Germany; 2 ERCO GmbH, 58507 Lüdenscheid, Germany

**Keywords:** Psychology, Engineering, Mathematics and computing, Optics and photonics

## Abstract

From the beginning of the $21\textrm{st}$ century until today, the demand for lighting systems includes not only visual parameters (brightness, contrast perception, color quality), but also non-visual parameters. It is necessary to define the new non-visual parameters for the realization of the new concept of Human Centric Lighting (*HCL*) or Integrative Lighting. As a contribution to this approach, many international research groups have tried to quantify the non-visual parameters such as Circadian Stimulus by Rea et. al. in USA ($CS_{2018}$, $CS_{2021}$), Melanopic Equivalent Daylight ($D_{65}$) illuminance, *mEDI* of the CIE S 026/E:2018 or the latest formula by Giménez et al., for the nocturnal melatonin suppression. Therefore, it is necessary to analyze the correlation between these non-visual metrics and brightness metrics such as the equivalent luminance of Fotios et al., or the latest brightness model of TU Darmstadt so that scientists, lighting engineers and lighting system users can correctly apply them in their work. In this context, this paper attempts to investigate and analyze these correlations between the three metric groups based on the database of 884 light sources of different light source technologies and daylight spectra. The obtained results show that the latest Circadian Stimulus model of Rea et. al. $CS_{2021}$ with the improvement of Circadian Light $CL_{A,2021}$ ($CL_{A\,2.0}$) has solved the disadvantage of $CS_{2018}$, especially for the interrupted point between warm and cold white (about $3710\,K$) or the junction between negative and positive signal of the opponent channel ($B -(L+M)$). Moreover, these three metrics of the three research groups contain a high correlation coefficient, so that one metric can be transformed by linear functions to the other two parameters.

## Introduction

Lighting research and vision science have a long history, accompanied by the dynamic evolution of light source technologies from thermal radiators such as tungsten and halogen incandescent lamps, to discharge light sources such as mercury, sodium, and fluorescent lamps, to the current technology of white and colored *LEDs* (Light Emitting Diodes)^1–6^. At each stage of this historical evolution, different visual tasks and visual metrics were defined to meet specific social, visual, and industrial needs. In the early decades of the $20\textrm{th}$ century, visual performance, including contrast vision, reaction time, visual acuity, and glare, was the focus of scientific and technological considerations. Thus, photometric quantities such as illuminance, luminance, uniformity, and glare index were defined using the $V(\lambda )$ function, the spectral luminous efficiency function for daytime vision^7,8^. With the development of fluorescent lamps and metal halide lamps, with more possibilities to modify the lamp spectra, several aspects of color quality such as color rendering index (*CRI*), correlated color temperature (*CCT*) and chromaticity of white light have been addressed in scientific literature and also in regulations for practical lighting^9–11^. With the continuous improvement of *LED* technology, new aspects of color quality at higher cognitive levels, such as color preference, color memory or color saturation, have been scientifically studied. These aspects have been introduced in practical applications, e.g. museum lighting^12–15^.

With the discovery of intrinsically photosensitive retinal ganglion cells (*ipRGCs*) containing melanopsin pigments^16–18^, two main lines of research on non-visual *ipRGC*-based effects have emerged. On the one hand, in lighting and sleep research, experiments have been conducted in laboratories or real-world settings (e.g., nursing homes, hospitals, schools, offices) to investigate the relationship between light intensity, spectrum, time, duration of light treatment, and non-visual outcomes (e.g., attention, sleep quality, alertness) using conventional photometric and colorimetric parameters such as vertical illuminance in lx, luminance in cd/$m^2$, color temperature, and spectral irradiance distributions^16–24^.

Since 2005, there has been a lot of research and discussion about how the receptor signals (rods, cones, and *ipRGCs*) combine to produce the non-visual effects of light. Section “The 2018 Circadian Stimulus ($CS_{2018}$) model” of this article describes the circadian stimulus (*CS*) models in the $CS_{2018}$ and $CS_{2021}$ versions of Rea et al.^1,2,25^. Melanopic Equivalent Daylight Illuminance (*mEDI*, in lx) has been introduced according to the *CIE* publication^3^. These two metrics, *CS* and *mEDI*, are currently proposed for scientific discussion worldwide. They are also subject to further investigation.

From a physiological point of view, these two metrics represent different opinions on how to define the quantities for non-visual effects. According to the above mentioned *CIE* publication, the non-visual effects should be based on the signals of the *ipRGCs*, which express the effect of the melanopsin pigments, so that the *mEDI* metric can be used for the calculation and evaluation of lighting systems regarding the aspect of their non-visual effects. The *CS* model of Rea et al, in the 2005 and 2018 versions^1,2^, is based on the idea that the non-visual effects come either from the *ipRGC* channel (if the opponent channel signal $(b - y \le 0)$, i.e. the blue channel has a weaker signal than the ($L+M$) or yellow channel, for example in the case of warm white light sources), or a combination of the *ipRGC* channel and the signals of the combinations of the ($L+M$), *S* cone and rod channels if ($b - y > 0$). The cases ($b - y \le 0$) and ($b - y > 0$) correspond to white light correlated color temperatures (*CCTs*) of, empirically, about ($CCT \le 3710\,K$) and ($CCT> 3710\,K$), respectively.

In recent years, roughly between 2018 and 2022, numerous international scientific discussions and analyses have been conducted to specify the correct metric for the non-visual effects, using data sets from experiments by different research groups on nocturnal melatonin suppression as a validation basis. The following important research results were achieved during this period:

1. Improvements of the *CS* model (versions 2005 and 2018) in the two years 2020 and 2021, taking into account the exposure time *t* (in hours) and the visual field, and modeling the contributions of the *ipRGC* channel, the *S* cones, the rods and the ($L + M$) channel with improved terms, for both cases ($b - y > 0$) and ($b - y \le 0$)^2,25^. This improved formulation was validated using data sets for melatonin suppression^2^ and is described in the present article.
2. Publication on *“Recommendations for daytime, evening, and night-time indoor light exposure to best support physiology, sleep, and wakefulness in healthy adults”* by a group of sleep researchers and neurophysiologists^26^.
3. Based on the analysis of experimental data on nocturnal melatonin suppression and using *mEDI* as an input metric for non-visual effects, Giménez et al.^4^ defined a formula to predict melatonin suppression with exposure time and pupil dilation as additional parameters. This new metric was found by a machine learning method and is a non-linear transformation of the *mEDI* metric. This formula, now called the Gimenez formula in this article, is compared to the *CS* metric in its 2021 version.

Parallel to the dynamic and intensive development of metrics for non-visual effects of light on humans, the development of metrics for brightness and visual clarity has experienced a renaissance with the development of quasi-monochromatic and phosphor-converted white *LEDs* with different correlated color temperatures and chromaticity coordinates. This new discussion started at the end of the $20\textrm{th}$ century by Fotios and Levermore^5^, with the development and analysis of new psychophysical methods in 2012^27^, and has been continued by the authors of the present article^6,28,29^. This renaissance could be explained by the fact that the brightness of white *LED* light sources of the same luminance but different spectra (different correlated color temperatures, chromaticity and color saturation enhancements) are perceived at different brightness levels. The root of these perceptual differences can be argued as the luminance or the signal of the luminance channel ($L + M$) is not solely responsible for the brightness perception, which includes additional contributions from rods, S cones, opponent channels^5,30–32^ and the *ipRGC* channel^33,34^. This argument is demonstrated in the brightness experiments of the PhD thesis of Pepler^35^.

With the above considerations in mind, the non-visual effects of light can be modeled either with a combination of signals from the *ipRGC* channel, the *S* cones, the ($L + M$) channel, and the rod channel ($CS_{2021}$ model), or with the *ipRGC* signals alone (*mEDI*, *CIE* publication S 026:2018^3^). Similarly, a brightness metric (denoted by *M*) can be constructed by using an exponential function of the ($L + M$) signal, the *ipRGC* signal, and the *S* cone signal^6^. From the point of view of lighting research and engineering, the following research questions arise:

1. Is there a reasonable and usable correlation between the melanopic equivalent daylight illuminance (*mEDI*) and the circadian light $CL_A$ in the *CS* models in the 2018 and 2021 versions; as well as the equivalent luminance $L_{eq}$ according to Eq. 1 of Fotios and Levermore^5^ (see section “Brightness perception and modeling” of this article) for brightness perception?
2. Is there also a useful correlation between the values of the $CS_{2021}$ metric, the Gimenez values for melatonin suppression, and the brightness perception metric according to the brightness metric in^6^?
3. What is the difference between the $CS_{2021}$ metric values and the Gimenez melatonin suppression values for the same light source spectra? Is this difference acceptable for practical use of these metrics?

If there is useable correlation between the different metrics and the difference between them is small enough to be in an acceptable range then a converting formula can be developed to transform one metric to the other with sufficient accuracy so that lighting researchers, sleep researchers or lighting engineers can design and evaluate a lighting system with several metrics recommended today although these metrics were established from different human physiological viewpoints. In the next sections, the brightness metric^6^, the model $CS_{2021}$ of Rea et al.^2^ and the formula of Giménez et al.^4^ will be presented before the correlations and relationships between the metrics for the non-visual effects and brightness will be described based on a calculation of 884 measured light source spectra.

## Brightness perception and modeling^5,6,36,37^

Over the past six decades, many research studies have been conducted using colored and conventional white light luminance experiments and modeling. The most important models are those of Guth et al.^38^ Ikeda et al.^39^, Kokoschka et al.^40^, Nakano et al.^32^, Palmer^41^, Ware and Cowan^42^, which led to a summary paper of the CIE (International Commission on Illumination) in^43^, which tested all models until 2001. All models included in this fundamental paper considered the contributions of the opponent channels ($L - M$) and ($S - (L + M)$) indirectly by implementing the chromaticity *x* and *y* into a joint function with the luminance of the achromatic signal ($L + M$). In a dissertation on photopic brightness in indoor lighting in 2017, Pepler^35^ varied the spectra of polychromatic white light sources and the luminances in the photopic range on a homogeneous and diffusely reflecting wall in a real room without daylight incidence and found in a comprehensive psychophysical experiment that under the defined test conditions with white light, the most consistent model corresponding to the subjective evaluations of the test subjects is a model by Fotios et al. from 1998^5^, in which the so-called equivalent luminance ($L_{eq}$) can be defined according to Eq. (1). This model divides the signal of the *S*-cones (*S*) by the signal of the *V*($\lambda$) function and then calculates a metric to the power of 0.24, see Eq. (1).

1 $$\begin{aligned} L_{eq} = L_v \cdot (S/V)^{0.24} \end{aligned}$$

In Eq. (1), the exponent of photopic luminance ($L_v$) remains 1.0, i.e., luminance remains uncompressed. To calculate the signals *S* or *V*, the relative spectral radiant flux of the light source must be multiplied by the spectral sensitivity function of the *S*-cones or by the *V*($\lambda$) function, respectively, and this product must be integrated over the visible wavelength range.

With the discovery of a new type of ganglion cells, the intrinsically photosensitive retinal ganglion cells (*ipRGCs*), described in several scientific publications, including Hattar et al. in^44^, some research has been conducted to answer the question whether ipRGC signals would also contribute to the perception of lightness in the photopic visual field. According to recent neurophysiological studies, there are some reasons to assume that *ipRGCs* interact with the visual channels in at least two different ways (see Zele et al.^33^):

1. $M_4$-subtype *ipRGCs* project to the LGN and contribute to human light perception (see Brown et al.^45^).
2. a group of $M_1$-subtype *ipRGCs* establish signaling connections with upstream dopaminergic amacrine cells. Luminance signals can be transmitted to the outermost sublamina of the inner plexiform layer, influencing the state of light adaptation (see Prigge et al.^46^).

As a result, studies by Zele et al.^33^ in 2018 and Yamakawa et al.^34^ in 2019 had found *ipRGC* signals in brightness perception. In 2015, Bullough et al.^36^ established a model for brightness that considers the contribution of the luminance channel *V*($\lambda$), *S*-cone *S*($\lambda$), and *ipRGC* (melanopsin, *Mel*($\lambda$)), which is described in Eq. (2).

2 $$\begin{aligned} B_2= V(\lambda )+ 0.6 \cdot g \cdot S(\lambda ) + 0.5 \cdot Mel(\lambda ) \end{aligned}$$

The *S*-cone contribution multiplier *g* in Eq. (2) depends on the level of adaptation and increases as a function of light level. In the Bullough et al.^36^ model in Eq. (2), the contributions of the luminance channel, *S*-cones, and *ipRGC* signals are integrated as a linear function into the brightness metric $B_2$. Brightness perception was analyzed and modeled in^6^ based on psychophysical experiments performed by the authors of the present article. For these experiments, 25 absolute spectra of multiple LED combinations (white LEDs and colored LEDs) with 5 different correlated color temperatures between 2700 and 10,000 K and 5 horizontal illuminances between 45 and 2000 lx with a relatively high color rendering index in the range $89 \le \text {IES TM-30-20 } R_f \le 93$ were used. The resulting brightness model is shown in Eq. (3) (TUD stands for “*TU Darmstadt*”).

3 $$\begin{aligned} M_{TUD,VT2023}=8.9974 \cdot \left[ E_v^{0.2629} \left( S^{0.074} + 0.5 \cdot G^{0.0424}\right) \right] -1.3307 \end{aligned}$$

This model (Eq. 3) contains the combination of an illuminance term compressed with the power function $E_v^{0.2629}$ and two terms with the compressed *S*-cone and*ipRGC* signal. The optimization based on the experimental data had also shown that, from a mathematical point of view, the contribution of the *S*-cones and the *ipRGC* channel is crucial. It was the intention of the model builders to present this brightness model for lighting applications in the photopic range with white light.

In the publication by Besenecker and Bullough in 2017 (see^37^), a carefully conducted brightness experiment was described. The two light sources to be compared could have near chromaticity with two different spectra (light source Amber1 with *S*-cone/photopic ratio of 0.27 and melanopsin/photopic ratio of 0.14 and light source Amber2 with *S*-cone/photopic ratio of 3.29 and melanopsin/photopic ratio of 1.13) and the experiments were performed at the two illuminances of the reference light source of 6.3 lx (mesopic range) and 108 lx (photopic vision). In Table 1 below, the mean perceived illuminance of the test light source by ten subjects is shown for the case where the illuminance of the reference light source (Amber 1 or Amber 2) is 108 lx, which produces an equivalent brightness. This table also shows the predictions according to Bullough’s model $B_2$ and the model $M_{TUD,VT2023}$. The two pairs of model brightness values according to model $M_{TUD,VT2023}$ for the two illuminances of the reference and test light sources in the case of the same brightness judged by ten subjects in the experiment mentioned above show a relative difference of $+12.3\%$ or $-10.5\%$. Both the $B_2$ model and the $M_{TUD,VT2023}$ model predictions are reasonably accurate for practical indoor lighting applications.

**Table 1:** Average equiluminance results at 108 lx for the reference light source (the data in the first four columns are after Table 5 in^37^).

	Light level reference (ref.) (lx)	Test light source (T. L. S.)	$B_2$ - prediction	Predicted Brightness (P. B.) $M_{TUD,VT2023}$	P. B $M_{TUD,VT2023}$	Re. B. Diff.in % $\Delta M_{TUD, VT2023}$
Amber 1 as [ref. L. S.]	108	94 SEM 5.7	98	[*For ref. L. S.*] 55.32	[*For T. L. S.*]62.15	+ 12.3
Amber 2 as [ref. L. S.]	108	125 SEM 5.1	119	[*For ref. L. S.*] 65.125	[*For T. L. S.*] 58.27	− 10.5

## Circadian stimulus models ($CS_{2018}$^1^ and $CS_{2021}$^2^)

The concept of Rea et al., covering the models $CS_{2005}$, $CS_{2018}$ or $CS_{2021}$ is based on the design of a phototransduction circuit which regards the following mechanisms:

- The phototransduction of photoabsorption, signal generation and conversion into a frequency-coded form, and the processing of the signals of the different channels (*LMS*—cones, rods and *ipRGCs*) exhibit subadditivity. Additivity is assumed, e.g., in the definition of illuminance or luminance with the $V(\lambda )$ function or the *mEDI* metric, when the spectral sensitivity of the receptor system is multiplied by the spectral radiance or spectral irradiance of the incident radiation and all effects at each wavelength between 380 nm and 780 nm can be integrated by summation to the final effect of the total polychromatic radiation. No signal reduction is expected. In contrast, a possible subadditivity occurs when the effect at a wavelength $\lambda _1$ is reduced when interacting with radiation of a certain wavelength $\lambda _2$. In neurobiology, subadditivity can be explained if the neural circuit for phototransduction contains a spectral opponent channel. In vision, two spectral opponent channels are known, a ($L - M$) channel and a ($S - (L + M)$) channel. In the context of non-visual effects, the spectral opponent channel ($S - (L + M)$) (also referred to as [d=TUD]$b - y$ $B - Y$) is taken into account. This is an important difference between the *CS* conception and the conception of the *mEDI* metric, which is defined by the *CIE*^3^ and assumes additivity of the nonvisual pathway. However, subadditivity was found to be essential in the experiments of Figuiero et al.^47,48^.
- The *CS* concept followed the idea that a non-visual effect consists of two components, a spectral component and a quantity component. The spectral [d=TUD]sensitivity functioncomponent, denoted by the circadian light $CL_A$ which will be described later, expresses the spectral generation of a stimulus at different receptors and channel systems (*LMS*-cones, rods, *ipRGC*) at a certain state of the spectral opponent channel ($(B - Y > 0)$ or $(B - Y \le 0)$). The definition of the *mEDI* metric does not distinguish between cases.
- The quantity component in the models from $CS_{2005}$, $CS_{2018}$ up to the model $CS_{2021}$ takes into account the exposure time, the characteristic of the visual field due to the spatial distribution of the *ipRGC* receptors on the retina, and the absolute magnitude of the circadian light value $CL_A$.
- A conversion from $CL_A$ to the circadian stimulus *CS* in the model versions $CS_{2005}$ and $CS_{2018}$ was based on the data sets of Thapan^17^ and Brainnard^16^ with quasi-monochromatic stimuli for nocturnal melatonin suppression. It has been improved and validated in 2021 by data sets from a variety of research groups. Therefore, the *CS* metric is also valid for lighting design processes for both evening and nighttime lighting.

### The 2018 circadian stimulus ($CS_{2018}$^1^) model

This model is implemented in two steps:

$1{\rm st}$ step: Establish circadian light $CL_A$ (denoted as $CL_A\,1.0$):

4 $$\begin{aligned} \mathrm {CL_A}={\left\{ \begin{array}{ll} 1548 \cdot \left[ \int M_{c\lambda }E_{\lambda } d\lambda + a_{b-y}\left( \int \frac{S_{\lambda }E_{\lambda } d\lambda }{mp_{\lambda }}d\lambda - k \int \frac{V_{\lambda }E_{\lambda } \cdot d\lambda }{mp_{\lambda }}\right) -a_{rod} \left( 1-e^{\frac{-\int V'_{\lambda }E_{\lambda } d\lambda }{RodSat}}\right) \right] &{} \text {if } \left( \int \frac{S_{\lambda }E_{\lambda }d\lambda }{mp_{\lambda }} - k \cdot \int \frac{V_{\lambda }E_{\lambda }d\lambda }{mp_{\lambda }}\right) > 0 \\ 1548 \cdot \left[ \int M_{c\lambda }E_{\lambda } d\lambda \right] &{} \text {if } \left( \int \frac{S_{\lambda }E_{\lambda }d\lambda }{mp_{\lambda }} - k \cdot \int \frac{V_{\lambda }E_{\lambda }d\lambda }{mp_{\lambda }}\right) \le 0 \end{array}\right. } \end{aligned}$$

In Eq. (4), the symbols have the following meanings:

- $CL_A$: Circadian Light where the subscript “*A*” designates a numerical equivalence of $CL_A = 1000$ (photopic) lx for CIE illuminant A.
- $E_{\lambda }$: Light source spectral irradiance.
- $M_{c\lambda }$: melanopsin sensitivity (corrected for crystalline lens transmittance, after Wyszecki and Stiles^49^).
- $$k = 0.2616$$.
- $S_{\lambda }$: S cone fundamental (Smith and Pokorny^50^).
- $$a_{b-y} = 0.7$$.
- $mp_{\lambda }$, macular pigment transmittance (after Snodderly et al.^51^).
- $$a_{rod} = 3.3$$.
- $V_{\lambda }$: Photopic luminous efficiency function (Commission Internationale de l’Éclairage^52^]).
- $$RodSat = 6.5 \textrm{W m}^{-2}$$.
- $V'_{\lambda }$: Scotopic luminous efficiency function (Commission Internationale de l’Éclairage^52^).

$2\textrm{nd}$ step: Conversion of the $CL_A$ value to Circadian Stimulus $CS_{2018}$.

The method of Rea et al., transforms the circadian effective light $CL_A$ with the help of Eq. (5) into a so-called “*circadian stimulus CS*”, which is proportional to the melatonin suppression in %. For example, a value of $CS = 0.4$ corresponds to a nocturnal melatonin suppression of 40 % compared to the pre-irradiation state.

5 $$\begin{aligned} CS=0.7-\dfrac{0.7}{1+\left( \frac{CL_A}{355.7}\right) ^{1.1026}} \end{aligned}$$

From the mathematical point of view, this logistical function in Eq. (5) shows that the $CS = 0.7$ or the nocturnal melatonin suppression of 70 % is reached if the Circadian Light term $CL_A$ is already very high. A higher *CS* value (higher than 0.7) is not possible. This is also a subject of the questions to be discussed later in relation to the formula of Giménez et al.^4^ in section “The formula of Giménez et al., for nocturnal melatonin suppression”. This *CS* value is valid for the exposure time of 1 h during the early biological night. Model values were validated by means of a dataset with 13 polychromatic light sources with the correlation coefficient of $r^2 = 0.69$^2^.

### The 2021 circadian stimulus ($CS_{2021}$^2^) model

The *CS* model $CS_{2018}$ has been used for a long time in some research groups and partly also by the US and international lighting industry with recognized advantages and clear deficits. According to the analysis of the authors of the present article, the boundary between “*cool*” and “*warm*” white polychromatic light sources in this aspect turned out to be about 3400–3710 K. According to^2^, the two steps for building the improved *CS* model 2021 were as follows:

$1\textrm{st}$ step: Establish a new for formula for circadian light $CL_A$ (denoted as $CL_A\,2.0$):

6 $$\begin{aligned} \mathrm {\textit{CL}_A\,2.0}= & {} {\left\{ \begin{array}{ll} 1548 \cdot \bigg [\int M_{c\lambda }E_{\lambda } d\lambda - a_{rod1}\left( \int \frac{V'_{\lambda }E_{\lambda } d\lambda }{\int V_{c\lambda }E_{\lambda }d\lambda + g_1 \int S_{c\lambda }E_{\lambda }d\lambda }\right) \cdot \left( 1-e^{\frac{-\int V'_{\lambda }E_{\lambda } d\lambda }{RodSat}}\right) + a_{b-y} \left( \int S_{c\lambda }E_{\lambda } d\lambda -k \int \frac{V_{\lambda }E_{\lambda } \cdot d\lambda }{mp_{\lambda }}\right) \\ - a_{rod2}\left( \int \frac{V'_{\lambda }E_{\lambda } d\lambda }{\int V_{c\lambda }E_{\lambda }d\lambda + g_2 \int S_{c\lambda }E_{\lambda }d\lambda }\right) \cdot \left( 1-e^{\frac{-\int V'_{\lambda }E_{\lambda } d\lambda }{RodSat}}\right) \bigg ]&{} \text {if } (b-y) > 0\\ 1548 \cdot \bigg [\int M_{c\lambda }E_{\lambda } d\lambda - a_{rod1}\left( \int \frac{V'_{\lambda }E_{\lambda } d\lambda }{\int V_{c\lambda }E_{\lambda }d\lambda + g_1 \int S_{c\lambda }E_{\lambda }d\lambda }\right) \cdot \left( 1-e^{\frac{-\int V'_{\lambda }E_{\lambda } d\lambda }{RodSat}}\right) \bigg ] &{} \text {if } (b-y) \le 0 \end{array}\right. } \end{aligned}$$

7 $$\begin{aligned} b-y= & {} \left( \int {S_{c\lambda }E_{\lambda }d\lambda } - k \cdot \int V_{c\lambda }E_{\lambda }d\lambda \right) \end{aligned}$$

8 $$\begin{aligned} V_{c\lambda }= & {} \frac{\frac{V_{\lambda }}{mp_{\lambda }}}{max \left( \frac{V_{\lambda }}{mp_{\lambda }}\right) } \end{aligned}$$

9 $$\begin{aligned} S_{c\lambda }= & {} \frac{\frac{S_{\lambda }}{mp_{\lambda }}}{max \left( \frac{S_{\lambda }}{mp_{\lambda }}\right) } \end{aligned}$$

with:

- $$k = 0.2616$$.
- $E_{\lambda }$: Light source spectral irradiance.
- $$a_{b-y} = 0.21$$.
- $M_{c\lambda }$: melanopsin sensitivity (corrected for crystalline lens transmittance, after Wyszecki and Stiles^49^).
- $$a_{rod1} = 2.3$$.
- $S_{\lambda }$: S cone fundamental (Smith and Pokorny^50^).
- $$a_{rod2} = 1.60$$.
- $mp_{\lambda }$, macular pigment transmittance (after Snodderly et al.^51^).
- $$g_1 = 1.00$$.
- $V_{\lambda }$: Photopic luminous efficiency function (Commission Internationale de l’Éclairage^52^])
- $$g_2 = 0.16$$.
- $V'_{\lambda }$: Scotopic luminous efficiency function (Commission Internationale de l’Éclairage^52^).
- $$RodSat = 6.5\,\textrm{W m}^{-2}$$

$2\textrm{nd}$ step: Conversion of $CL_A\,2.0$ value to Circadian Stimulus $CS_{2021}$.

Compared to the model version $CS_{2018}$, the basic structure of the version 2021 with the logistic function remains unchanged with the exponent 1.1026 and the half saturation constant 355.7 (with the *CS* value of 35% as half of the maximum possible melatonin suppression, 70%). Two new factors have been implemented, the exposure time *t* (in hours) of 0.5 and 3 h and the factor *f* describing the spatial distribution of the circadian light exposure.

10 $$\begin{aligned} CS_{t,f}=0.7-\dfrac{0.7}{1+\left( \frac{CL_{A,2.0}\cdot t \cdot f}{355.7}\right) ^{1.1026}} \end{aligned}$$

Regarding the factor *f* in three different viewing modes, Rea et al. defined three values^2^:

- For a full visual field (a Ganzfeld): $f = 2.0$.
- For a central visual field (e.g. with a light box on a desk): $f = 1.0$.
- For a superior visual field (e.g. from ceiling mounted down-light luminaires): $f = 0.5$.

However, the above definition of viewing conditions is not precise for practical lighting applications. For the purposes of numerical analysis in this article, *f* is set to 1.0.

## The formula of Giménez et al., for nocturnal melatonin suppression^4^

The numerical study of Giménez et al.^4^ pursued similar intentions as the *CS* models with the following research conception:

1. The authors of this study aimed to build a metric with *mEDI* (Melanopic Equivalent Daylight ($D_{65}$) illuminance) as the starting parameter and extended the analysis to include the contributions of *LMS* cones based on a correlation analysis of 29 different data sets of nocturnal melatonin suppression published in scientific papers.
2. The co-parameters were the exposure time and the pupil state of the subjects during the experiments (with or without pupil dilation). The metric to be found should be a metric for predicting nocturnal melatonin suppression similar to the above mentioned $CS_{2021}$ model of Rea et al.

The data analysis was based on the Random Forest (*RF*) method, a machine learning approach to solving classification and regression problems. The model was constructed in two steps. In the $1\textrm{st}$ step, *mEDI* illuminance, photopic illuminance, rhodopic *EDI* (for rods), *L*-opic *EDI*, *M*-opic *EDI*, and *S*-opic *EDI* were subjected to separate correlation analyses at different exposure times for narrowband and polychromatic light spectra. From 21 to 10,000 lx, the *mEDI* metric showed the best correlation coefficients. The *S* cone *EDI* outperformed the *mEDI* metric only in the range below 21 lx.

With *mEDI* illuminance as the initial parameter, other components such as *LMS* cone signals, exposure time (duration), and pupil dilation were added to the set of input parameters, and a four-parameter logistic function was constructed and compared to the available data sets. The accuracy of the regression analysis was expressed as the root mean square error (*RMSE*). The optimal random forest model was the model with the lowest *RMSE* and the least number of predictors. In addition, the coefficient $r^2$ was also used. The results of this analysis can be summarized as follows:

- Adding *L* and *M* cones did not improve the model quality compared to the combination of *S* cones and *ipRGC* alone.
- The combination of *ipRGC* and *S* cones resulted in a higher correlation coefficient and a lower *RMSE* value compared to *ipRGC* alone (*mEDI*). The difference was rather small, so the Giménez research group decided to ignore the *S*-signal portion in their model.
- The logistic function model therefore includes *mEDI*, exposure time and pupil dilation, see Eq. (11).

11 $$\begin{aligned} Suppression_{melatonin}=\frac{0-100}{1+\frac{log_{10}(mEDI_{melanopic} \cdot 10^6)}{9.002-0.008 \cdot \Delta t_{expose} - 0.462 \cdot dil_{pupil}}} \end{aligned}$$

In Eq. (11), the symbols have the following meaning:

- $suppression_{melatonin}$= melatonin suppression (in %)
- $mEDI_{melanopic}$ = melanopic *EDI* (lx)
- $\Delta t_{exposure}$ = exposure duration (in minutes)
- $dil_{pupil}$ = pupil dilation applied: 0 = no, 1 = yes

Compared to the *CS* model of Rea et al., the value of melatonin suppression is up to 100% at infinite illuminance. In the opinion of the authors of the present article, the most important difference between the *CS* model in the version $CS_{2021}$ (or $CS_{2018}$) and the model of Giménez et al., is the aspect of value scaling. (Note that both models were built using regression methods for nocturnal melatonin suppression data. Both models have been validated using partially similar data sets from well-known research groups).

## Numerical analysis of the relationship between brightness and non-visual metrics

### Introduction

The brightness metrics and the nonvisual effects of light metrics described above can be grouped into the following categories:

- A set of linear metrics: Fotios’ model for equivalent luminance without signal compression (Eq. 1); circadian light $CL_A$ for model versions 2018 and 2021; and *mEDI* (which is also an input parameter of the logistic function of Giménez et al.).
- A group of nonlinear metrics such as *CS* ($CS_{2018}$ and $CS_{2021}$); the metric according to Giménez et al. (output parameter of the logistic function in Eq. (11); and the brightness model *M* of the authors of the present article, see Eq. (3).
- Since the *CS* model includes the case distinction between “*warm*” and “*cold*” white light ($$B - Y > 0)$$ or ($$B - Y \le 0$$), or empirically if ($$CCT\,>\,3710$$ K) or ($$CCT\,\le \,3710$$ K), the correlation with experimental data may depend on the type of light spectra actually used.
- Since the *CS* model (in the $CS_{2018}$ version) has been used for a long time by U.S. and international lighting science and industry, it is necessary to analyze the differences and correlations between the values of the $CS_{2018}$ and $CS_{2021}$ versions, as well as the “*warm*” and “*cold*” light categories.

### Correlation analysis method

**Figure 1:**
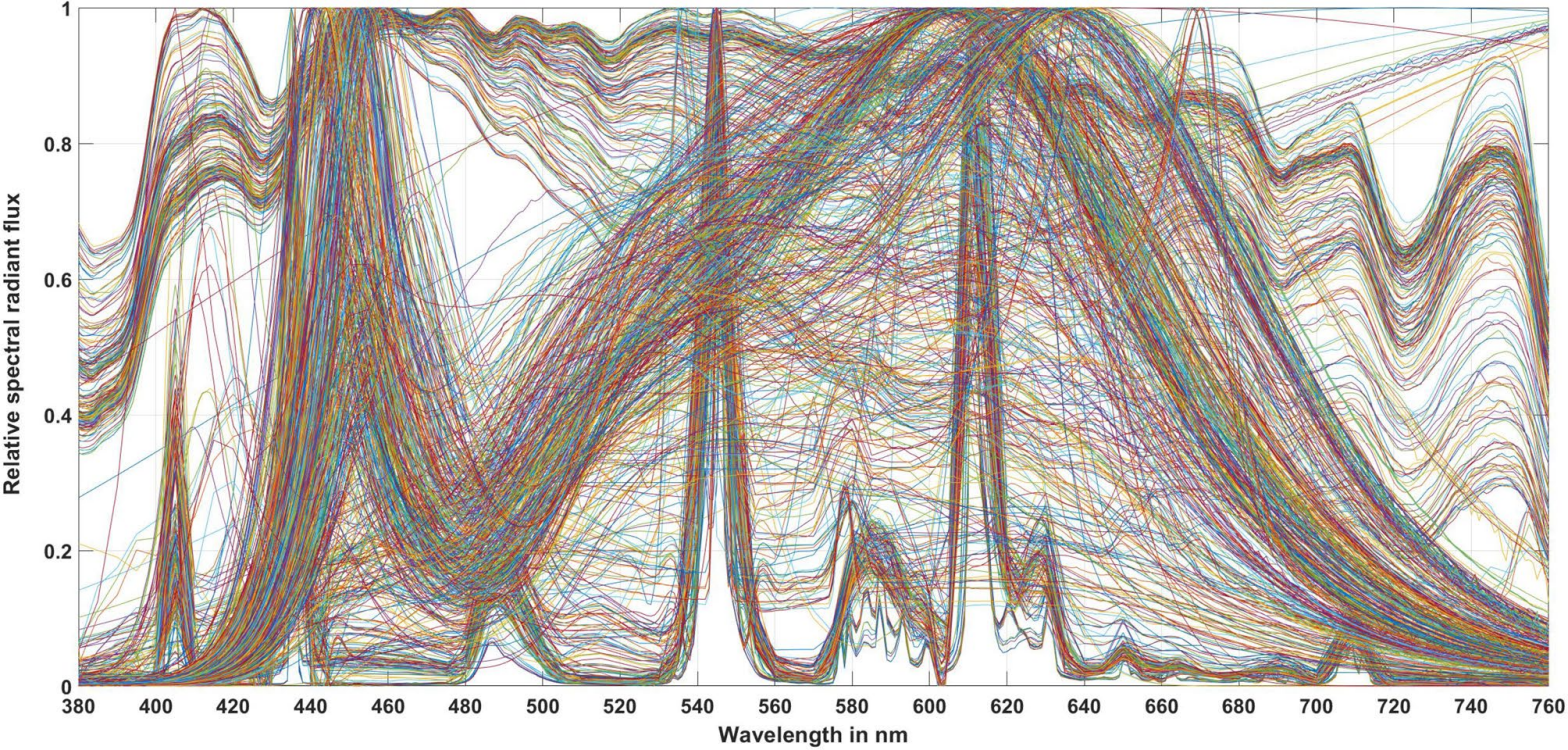
Spectra of 884 real measured light source spectra.

For this correlation analysis, several measured light source spectra were analyzed, see Table 2. This set of spectra includes real measured light sources of incandescent lamps (thermal radiators, 28 light sources), compact and linear fluorescent lamps (252 light sources), different types of LEDs (419 light sources), and 185 measured daylight spectra on a clear sunny day and a rainy cloudy day at different hours of the day in the city of the Technical University of Darmstadt (city of Darmstadt, Germany) at an amusement park for students. These 884 light source spectra are shown in Fig. 1.

**Table 2:** Light sources and their photometric and colorimetric quantities in the correlation analysis.

No.	Type of light sources and their parameter ranges 2201 $\le$ *CCT* $\le$ 17815 K; -1.467$\cdot 10^{-2}$ < *Duv* < 1.529$\cdot 10^{-2}$ 0.0797 < *z* < 0.4792; 80 < $CIE\,R_a$ < 100
1.	Conventional incandescent lamps + filtered incandescent lamps, 28 spectra
2.	Fluorescent tubes + Compact fluorescent lamps, 252 spectra
3.	LED lamps + LED luminaires, 419 spectra
4.	Daylight (measurements), 185 spectra

To calculate the values of the brightness metrics, the *CS* values, and the *mEDI* and Giménez values, the 884 spectra in Table 2 were converted into a set of absolute spectra at three fixed photopic vertical illuminance levels: 125 lx, 500 lx, and 750 lx, which cover the range of illuminance levels in practical indoor lighting applications. Brightness and non-visual parameters were then calculated at each of these three illuminance levels according to Eqs. (1)–(11).

### Relationship between linear brightness metrics and non-visual effect parameters

Table 3 shows the correlation coefficients between the linear brightness metrics and the non-visual effect parameters for all 884 light source spectra in Table 2 (regardless of their correlated color temperatures, i.e., including both the warm white and cold white spectra).

**Table 3:**
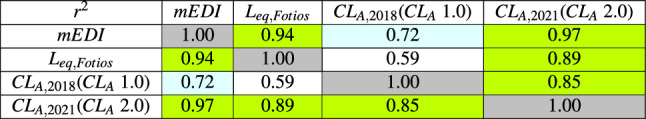
Correlation between the linear brightness metrics and the non-visual-effect parameters for all spectra in Table 2 (correlated color temperatures between 2201 and 17815 K).

The following can be seen from Table 3:

- The correlation between *mEDI*, $CL_{A,2021}$ ($CL_A$ 2.0) and equivalent luminance according to Fotios is high, with the following $r^2$ values:
  - 0.89 ($$L_{eq,Fotios}$$ vs. $$CL_{A,2021}$$ ($$CL_A$$ 2.0));
  - 0.94 (*mEDI*, vs. $L_{eq, Fotios}$); and
  - 0.97 ($$CL_{A,2021}$$ ($$CL_A$$ 2.0) vs. *mEDI*).
- The correlation coefficient between $CL_{A,2018}$($CL_A$ 1.0) and *mEDI* or $L_{eq, fotios}$ is much lower ($r^2$ equals 0.72 or 0.59).
- For all spectra between 2201 K and 17815 K, the correlation coefficient between $CL_{A,2018}$ ($CL_A$ 1.0) and $CL_{A,2021}$ ($CL_A$ 2.0) equals 0.85.

**Figure 2:**
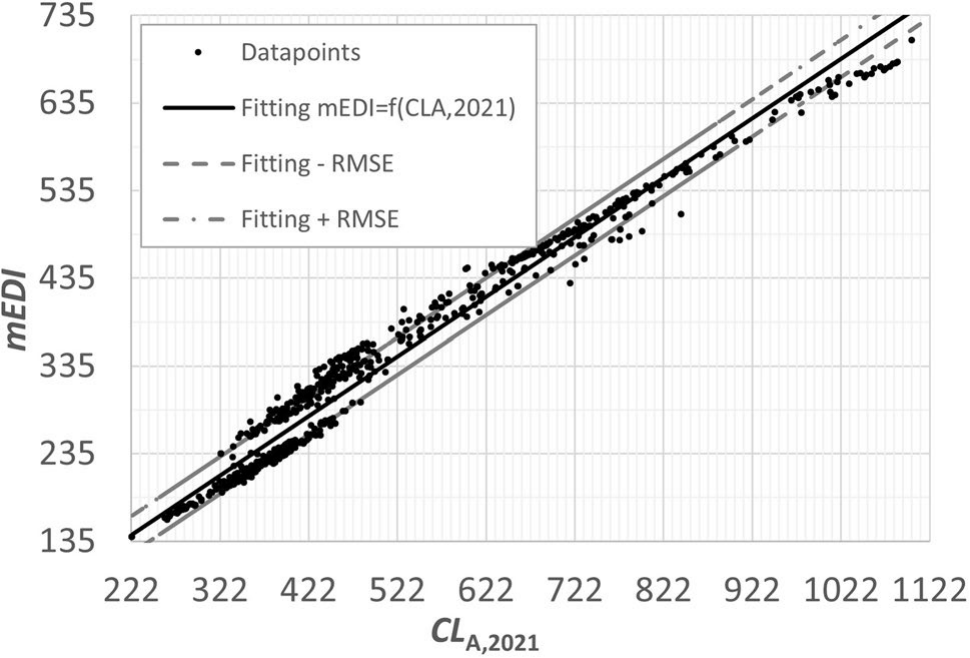
Correlation between *mEDI* and $CL_{A,2021}$ ( $CL_A$ 2.0) for all spectra in Table 2 between 2201 and 17815 K.

The $CL_{A,2021}$ ($CL_A$ 2.0) values tend to correlate very well with *mEDI* (see Fig. 2, *RMSE* = 21.25) and the equivalent luminance of Fotios, much better than with $CL_{A,2018}$($CL_A$ 1.0). For all spectra between 2201 K and 17815 K, a linear relationship was found between *mEDI* and $CL_{A,2021}$ ($CL_A$ 2.0), see Eq. (12) and Fig. 2.

12 $$\begin{aligned} mEDI=0.6792 \cdot CL_{A,2021}(\thicksim CL_{A,2.0})-8.3139 \end{aligned}$$

If only the spectra in the “*warm white*” region ($CCT \le 3710$ K, $B - Y \le 0$) are considered, a different picture emerges. These correlations are shown in Table 4.

**Table 4:**
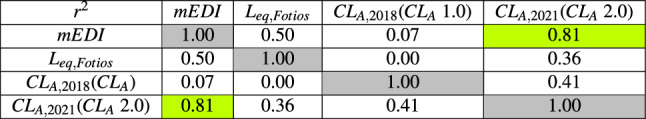
Correlation between linear brightness metrics and non-visual effect parameters for the correlated color temperature range $2200\,K\,\le \,CCT\,\le \,3710\,K$ (warm white light).

The following can be seen from Table 4:

- $CL_{A,2018}$($CL_A$ 1.0) correlates poorly with $CL_{A,2021}$($CL_A$ 2.0) ($r^2= 0.41$), with $L_{eq,Fotios}$ and with *mEDI*.
- $L_{eq,Fotios}$ correlates also moderately, rather poorly with *mEDI* and $CL_{A,2021}$($CL_A$ 2.0).
- *mEDI* and Circadian Light $CL_{A,2021}$($CL_A$ 2.0) exhibit a good correlation with $r^2 = 0.81$.

**Figure 3:**
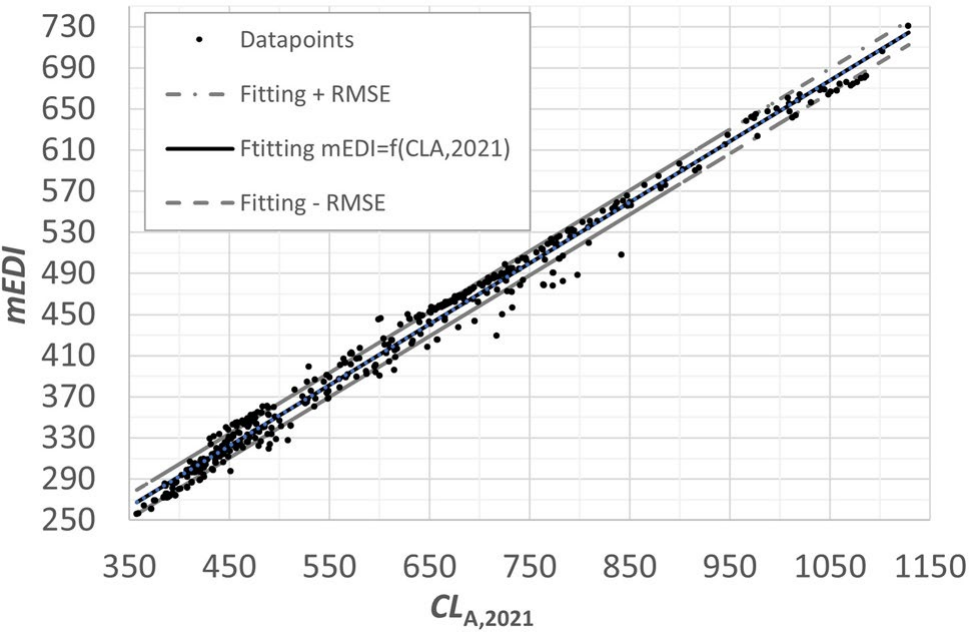
Correlation between *mEDI* and $CL_{A,2021}$ for the spectra in Table 2 between 3710 and 17815 K.

Finally, looking at the correlated color temperature range between 3710 K and 17815 K (neutral white and cold white illuminants), the correlation coefficients between all brightness metrics and non-visual metrics are very good, see Table 5. The values of $CL_{A,2021}$($CL_A$ 2.0) correlate very well with *mEDI*, $CL_{A,2018}$($CL_A$ 1.0) and $L_{eq,Fotios}$, see Fig. 3.

**Table 5:**
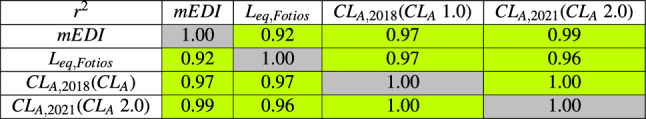
Correlation between linear brightness metrics and non-visual effect parameters for the correlated color temperature range $3710\,K<\,CCT\,\le \,17815\,K$ (neutral and cold white light).

A formula was derived for the range $3710 K < CCT \le 17,815 K$ with an *RMSE*-value of only 11.85, see Eq. (13).

13 $$\begin{aligned} mEDI=0.5924 \cdot CL_{A,2021}(\thicksim CL_{A,2.0}) + 55.5 \end{aligned}$$

From the practical point of view of lighting engineering, it must be emphasized that most buildings and rooms in the private and professional sectors will have lighting situations with *CCTs* higher than 3710 K during the daytime with daylight incidence (i.e. with windows), so that the conversion between *mEDI* and $CL_{A,2021}$($CL_A$ 2.0) according to Eq. (13) is of high importance.

The above results lead to the conclusion that the correlation between *mEDI* and the new version $CL_{A,2021}$($CL_A$ 2.0) is good for the warm white range (e.g. for evening applications) and very good for the range between 3710 K and 17815 K for indoor lighting in residential and commercial buildings (e.g. for offices, schools, supermarkets) as well as for outdoor daylight. The improvement from Circadian Light $CL_{A,2018}$($CL_A$ 1.0) to the new version $CL_{A,2021}$($CL_A$ 2.0) is significant.

### Relationship between non-linear brightness metrics and non-visual effect parameters

The correlation analysis for all 884 spectra between 2201 K and 17815 K gives in this case the results in Table 6. For this analysis, the exposure time of 1 h was chosen in the case of $CS_{2021}$ and for the formula of Giménez et al. (denoted as $Sups.Gim._{t=1h,pul.d.=0}$) and no pupil dilation was taken into account according to the usual illumination applications and viewing situations in practice.

**Table 6:**
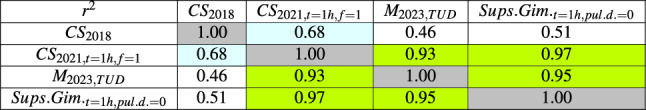
Correlation between the non-linear brightness metrics $M_{2023,TUD}$ and the *CS* model versions 2018–2021 as well as the Giménez metric for all spectra in Table 2 in the range $2201\,K\,\le \,CCT\,\le \,17815\,K$.

The following can be seen from Table 6:

- $CS_{2018}$ shows moderate correlations to $Sups.Gim._{t=1h,pul.d.=0}$ and brightness $M_{2023,TUD}$ ($r^2$ equals 0.51 and 0.46, respectively).
- $CS_{2021,t=1h,f=1}$ correlates very well with $Sups.Gim._{t=1h,pul.d.=0}$ and $M_{2023,TUD}$ ($r^2$ equals 0.97 and 0.93, respectively).
- The correlation between the brightness metric $M_{2023,TUD}$ and $CS_{2021,t=1h,f=1}$ and the Giménez metric is very good with $r^2$ = 0.93 and 0.95, respectively.

**Figure 4:**
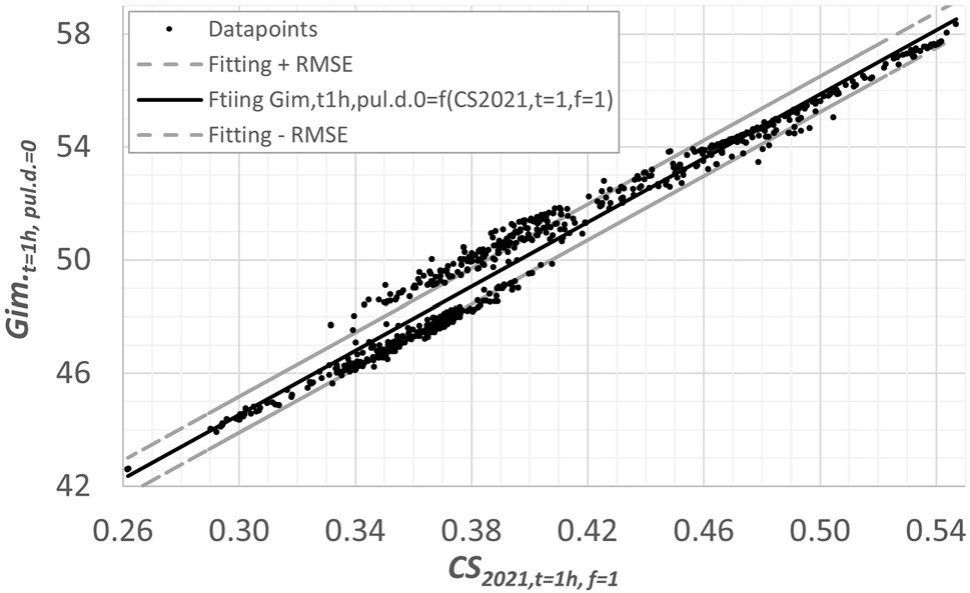
Relationship between the Giménez value and $CS_{2021,t=1h,f=1}$ for all spectra in Table 2 between 2201 and 17815 K.

Figure 4 shows the relationship between the Giménez values (exposure time 1 h, no pupil dilation) and the $CS_{2021,t=1h,f=1}$ values (exposure time 1 h, $f=1$) for a practically relevant range of values of $CS_{2021,t=1h,f=1}$ between 0. 26 and 0.54, corresponding roughly to the vertical illuminance between 280 and 1550 lx at the correlated color temperature of 4000 K. This relationship was modeled by the formula in Eq. (14) with $r^2$ of 0.97.

14 $$\begin{aligned} Sups.Gim._{t=1h,pul.d.=0} (in \%) =56.699 \cdot CS_{2021,t=1h,f=1} + 27.5 \end{aligned}$$

Table 7 shows the correlation coefficients in the case of the warm white range $CCT \le 3710\,K$. As can be seen from Table 7, the $CS_{2018}$ values do not correlate with $CS_{2021 ,t=1h,f=1}$, $Sups.Gim._{t=1h,pul.d.=0}$ (of the Giménez metric) and the brightness values according to the $M_{2023,TUD}$ formula. The values $Sups.Gim._{t=1h,pul.d.=0}$ of the Giménez metric correlate relatively well with the values of the brightness metric $M_{2023,TUD}$ ($r^2 = 0.62$) and especially with $CS_{2021,t=1h,f=1}$ ($r^2 = 0.85$).

**Table 7:**
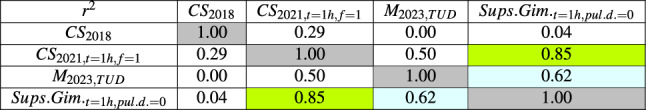
Correlation between the nonlinear brightness metric $M_{2023,TUD}$ and the *CS* model versions 2018–2021 as well as the Giménez metric for the warm white spectra in Table 2$(2201 K \le CCT \le 3710 K)$.

**Table 8:**
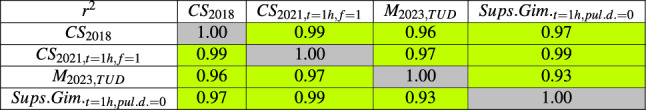
Correlation between the nonlinear brightness metric $M_{2023,TUD}$ and the *CS* model versions 2018–2021 as well as the Giménez metric for the neutral and cold white spectra in Table 2 ($3710 K < CCT \le 17815 K$).

Table 8 shows the correlation coefficients for the case of the wide range of correlated color temperatures between 3710 and 17,815 K. All metrics for brightness and non-visual light effects express a very good correlation with each other.

## Discussion and summary

In this article, the concept of non-visual parameters such as the circadian stimulus modes of the Circadian Stimulus by Rea et. al. in the USA ($CS_{2018}$^1^, $CS_{2021}$^2^), melanopic equivalent daylight ($D_{65}$) illuminance, *mEDI* of the CIE S 026/E: 2018^3^ and the latest formula of Giménez et al.^4^ for nocturnal melatonin suppression^4^ are briefly described to understand their structure and characteristics. Also, the equivalent luminance of Fotios et al.^5^ or the brightness of the TU Darmstadt^6^ are briefly introduced.

Then the calculations and analyses based on the databases of 884 light sources (26 conventional incandescent lamps and filtered incandescent lamps, 252 fluorescent tubes and compact fluorescent lamps, 419 LED lamps and LED luminaires, and 185 daylight from measurements) were implemented.

Summarizing the results of this article, $CS_{2021}$ is a significant improvement over $CS_{2018}$, which did not work in the warm white region ($CCT \le 3710\,K$). The correlation between the brightness metric $M_{2023,TUD}$, the Giménez metric (based on the *mEDI* metric) and $CS_{2021}$ based on $CL_{A,2021}(CLA\,2.0)$ is good or even very high. Consequently, these three metrics can be converted by linear formulas (see the equations Eqs. (12)–(14) with acceptable accuracy from an engineering point of view.

### Informed consent

All authors have read the submitted version of the manuscript.

## Data Availability

The datasets used and/or analysed during the current study available from the corresponding author on reasonable request.
